# The association between depressive and sleep symptoms for predicting incident disease onset after 6-year follow-up: findings from the English Longitudinal Study of Ageing

**DOI:** 10.1017/S0033291718001290

**Published:** 2018-05-29

**Authors:** Lydia Poole, Marta Jackowska

**Affiliations:** 1 Department of Behavioural Science and Health, University College London, 1-19 Torrington Place, London, WC1E 6BT, UK; 2 Department of Psychology, University of Roehampton, London, SW15 4JD, UK

**Keywords:** Depressive symptoms, disease onset, English Longitudinal Study of Ageing, physical illness, sleep

## Abstract

**Background:**

The independent effects of depressive symptoms and sleep problems for future physical illness risk have yet to be studied systematically across a variety of disease endpoints.

**Methods:**

We analysed data from 7395 participants (65.81 ± 9.39 years; 54.8% female) from the English Longitudinal Study of Ageing (ELSA). Baseline was wave 4 and participants were followed up for 6 years until wave 7. Sleep was measured using an adapted version of the Jenkins Sleep Problems questionnaire and depressive symptoms using the Centre for Epidemiological Studies Depression scale. Participants with the illness of interest at baseline [coronary heart disease (CHD), cancer, diabetes/high blood glucose, arthritis] were excluded from models predicting the onset of that illness at follow-up. Logistic regression was used, entering depressive symptoms and sleep problems simultaneously into models controlling for a wide range of covariates.

**Results:**

In fully adjusted models depressive symptoms predicted incident CHD (OR 1.11, 95% CI 1.04–1.20, *p* = 0.004) and diabetes/high blood glucose (OR 1.13, 95% CI 1.04–1.22, *p* = 0.002) independent of sleep problems; both depressive symptoms (OR 1.10, 95% CI 1.04–1.16, *p* = 0.002) and sleep problems (OR 1.14, 95% CI 1.02–1.26, *p* = 0.019) predicted incident arthritis.

**Conclusions:**

Sleep problems and depressive symptoms, and a combination of both, were differentially associated with physical illness onset 6 years later. Our findings highlight the importance of taking into account somatic and affective experiences when looking across a variety of different physical illnesses.

## Introduction

Depression in physical illness is highly prevalent (Moussavi *et al.*, 2007). Several good quality reviews have also shown that depression can lead to physical illness onset. For example, a meta-analysis of longitudinal studies showed depressed individuals to be at a 60% greater risk of developing type II diabetes than those without depression (Mezuk *et al.*, 2008). Depression has also been implicated in the aetiology of coronary heart disease (CHD) (Kuper *et al.*, 2002; Rugulies, 2002; Nicholson *et al.*, 2006), stroke (Pan *et al.*, 2011), cancer (Chida *et al.*, 2008), and dementia (Diniz *et al.*, 2013). However, the precise mechanism of this effect is unclear. Notably, the somatic dimension of depression symptomatology may be particularly important in understanding this association (Ormel and de Jonge, 2011; Poole *et al.*, 2011; Carney and Freedland, 2012).

One somatic symptom of depression that is also known to be independently associated with the disease is sleep. Several reviews have described the relationship between depression and sleep (Benca *et al.*, 1992; Tsuno *et al.*, 2005; Benca and Peterson, 2008). Depression is frequently accompanied by changes to sleep, and patients with major depressive disorder (MDD) are more likely than those without to experience problems with sleep onset, sleep maintenance and early morning awakenings (Benca and Peterson, 2008); these findings have been confirmed using objective readings of sleep collected via actigraphy and polysomnography (Tsuno *et al.*, 2005). However, despite sleep disturbance being a diagnostic characteristic of MDD, the relationship between depression and sleep is now considered bidirectional. For example, depression is highly prevalent in those with insomnia (Taylor *et al.*, 2005) and persons with insomnia are also at increased risk of developing a new-onset depressive episode compared with those without sleep problems (Riemann and Voderholzer, 2003; Baglioni *et al.*, 2011). Common biological pathways have been proposed to explain the link between sleep and depression including changes to sleep architecture, specifically rapid eye movement (REM) sleep, and neurobiological processes involving deficits in the monoaminergic and neuroendocrine systems (for a review see Baglioni and Riemann, 2012).

As alluded to above, poor sleep has been shown to be associated with chronic physical illness. A large multi-country study of over 42 thousand adults aged 50 years and older found elevated sleep problems in nine different physical illnesses, including angina, arthritis, diabetes and stroke (Koyanagi *et al.*, 2014). Sleep duration has been implicated as a risk factor for cancer in a large prospective cohort study (Gu *et al.*, 2016). However, less is known about the prospective relationship between sleep, depression and physical illness, specifically the role of depression and sleep problems for disease onset. One review that has attempted to address this issue examined the combined effect of depressive symptoms and sleep problems for cardiometabolic disease risk [i.e. cardiovascular disease (CVD) and diabetes] (Mezick *et al.*, 2011). These authors found evidence for sleep continuity (i.e. difficulties initiating and maintaining sleep) and depression being independently associated with CVD, however, findings for diabetes were inconclusive and further research was recommended. Furthermore, given the high prevalence of comorbidity in today's ageing population (Marengoni *et al.*, 2011; Garin *et al.*, 2016), more research is needed to investigate the role of depression and sleep for the onset of other chronic illnesses such as cancer and arthritis.

Several questions remain to be addressed. Firstly, to what extent do depression and sleep independently predict the onset of different physical illnesses? Secondly, are there interaction effects between depression and sleep symptoms such that an additive effect is more health damaging than either complaint alone? And thirdly, do similar patterns emerge across different physical illnesses or are there similar associations across conditions? Using nationally representative data from the English Longitudinal Study of Ageing (ELSA) (Steptoe *et al.*, 2013) we aimed to address these three research questions. This cohort of adults aged 50 years or older is pertinent to answer these research questions given the greater prevalence of physical illnesses in older adults (Manini, 2011). These were exploratory analyses, so no directional hypotheses were made.

## Methods

### Sample and study design

Data were taken from ELSA, a nationally representative general population study of adults aged 50 years and older living in England. Further details can be found elsewhere (Steptoe *et al.*, 2013). The sample is followed up every 2 years from 2002 onwards, with refresher samples being added at waves 3, 4 and 6. Wave 7 is the most recently completed phase of data collection. At every wave, participants complete a computer-assisted personal interview plus a self-completion questionnaire. On alternate waves, a nurse visit is conducted to allow for the collection of blood samples and objective assessments of physical function, such as body mass index (BMI). Nurse visits were only conducted on core sample members who had an interview in person.

**Fig. 1.**
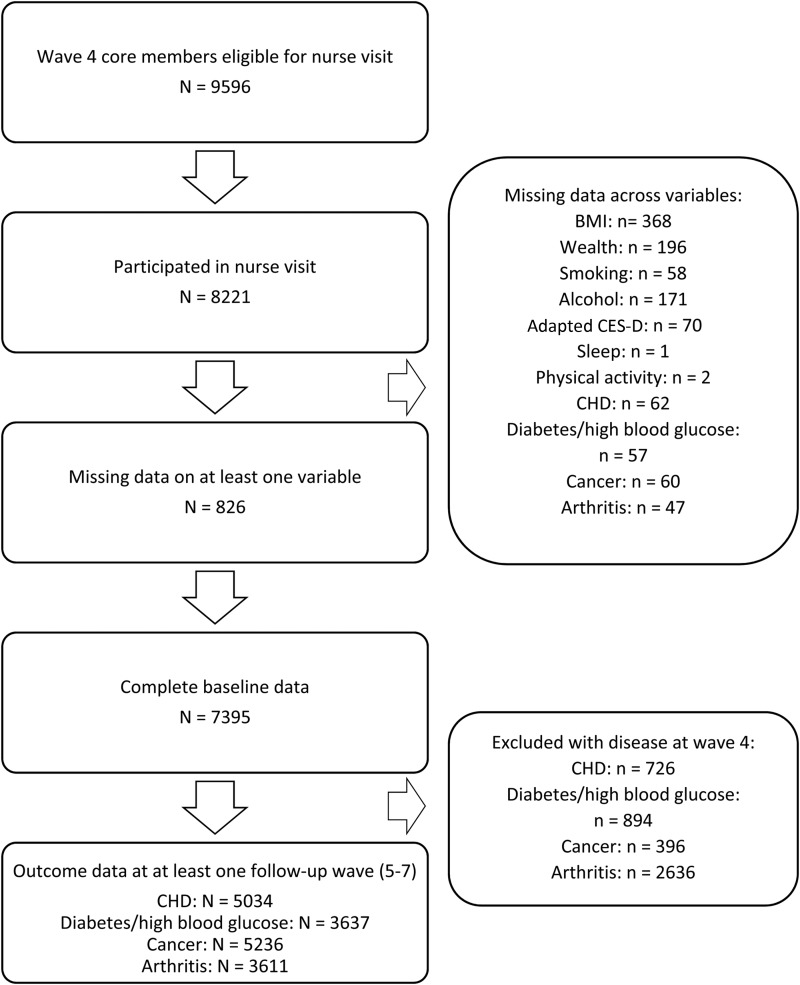
Flow diagram of sample.

This current paper reports data spanning 6 years, from wave 4 (2008/09) through to wave 7 (2014/15), of core members with nurse visit data. Wave 4 was selected as a baseline since this was the first point at which sleep data were collected. Complete case analysis was performed on a sample of 7395 participants (65.81 ± 9.39 years; 54.8% female) who provided data on independent and dependent variables and covariates. A flow diagram of how the sample was derived is provided in Fig. 1. Overall, compared with those excluded from these analyses, those included were more likely to be cohabiting (χ^2^ = 25.09, *p* < 0.001), from a higher wealth quintile (χ^2^ = 101.37, *p* < 0.001), have lower adapted (term explained in Section 2.2.1 below) CES-D scores (*t* = 6.63, *p* < 0.001), engage in more regular exercise (χ^2^ = 30.59, *p* < 0.001) and consume alcohol more frequently (χ^2^ = 88.90, *p* < 0.001). They were also less likely to have hypertension (χ^2^ = 4.43, *p* = 0.036). Specific Ns are reported for models relating to each incident illness.

### Measures

#### Depressive symptoms

Depressive symptoms were measured at baseline using the eight-item Centre for Epidemiological Studies Depression scale (CES-D). The CES-D measures symptoms that can be used to identify people at risk of depression, rather than clinical depression *per se* (Radloff, 1977). The psychometric properties of the eight-item version have been shown to be comparable with the original 20-item version (Steffick, 2000). For the purpose of analyses described here the sleep item was removed from the CES-D so as to avoid the issue of shared variance with the sleep problems measure, hence we term this measure an ‘adapted CES-D’. The Cronbach's alpha for the adapted CES-D in this study was 0.80. We computed a summary score by adding responses to all seven dichotomous questions (possible range: 0–7).

#### Sleep problems

Sleep problems were measured with three questions referring to the most frequent insomnia symptoms, including difficulties falling asleep, waking up several times a night and waking up in the morning feeling tired. These sleep items were derived from the Jenkins Sleep Problems Scale (Jenkins *et al.*, 1988). Participants answered these questions with regards to the past month. Items were rated on a 4-point Likert scale ranging from ‘not during the past month’ to ‘three of more times a week’. Scores were averaged (range 1–4) with higher scores indicating more sleep problems. The Cronbach's alpha at wave 4 for this sample was 0.60.

#### Incident chronic disease

Participants were shown a list of illnesses and asked to self-report any doctor diagnosed illnesses they had received. The incident chronic disease was defined as a positive report of CHD, cancer, diabetes/high blood glucose or arthritis by participants at waves 5, 6 and 7, excluding participants who reported that same chronic illness at wave 4 baseline. These diseases were selected based on their consistency of reporting across waves and due to their prevalence in ELSA. CHD was defined to include all cases of angina and myocardial infarction. To avoid self-reporting errors, arthritis was coded to include osteoarthritis, rheumatoid and arthritis, type not specified cases. The categorisation of diabetes/high blood glucose included both participant self-reports of the illness as well as a positive glycated haemoglobin (HbA1c) value. HbA1c was assessed from blood drawn from participants’ forearm during the nurse visits at waves 4 and 6. Participants who had a clotting or bleeding disorder and those on anti-coagulant medication did not provide blood samples. Fasting samples, defined as not eating or drinking for 5 h prior to blood draw, were collected where possible and when not otherwise contraindicated (e.g. >80 years, diabetic, frail or unwell, ever had a seizure). All blood samples were analysed at the Royal Victoria Infirmary laboratory in Newcastle upon Tyne, UK. A detailed description of blood analyses is available elsewhere (Sproston and Mindell, 2004). At wave 6, HbA1c was analysed in the International Federation of Clinical Chemistry units (mmol/mol) rather than the Diabetes Control and Complications Trial (%) units. For consistency wave 6 values were converted to % using the formula (XX/10.929) + 2.15. We used the classification of greater than 6.5% HbA1c to signify probable diabetes as recommended by the World Health Organisation (World Health Organization, 2011).

#### Covariates

Covariates were all measured at baseline. Sociodemographic variables include age, sex, and whether participants were married or cohabiting with a partner. Socioeconomic status was included in models as quintiles of net financial wealth, which refers to participants’ gross financial wealth with financial debt subtracted. Height and weight were collected during the wave 4 nurse visit and BMI was derived using the standard formula (kg/m^2^). Whether or not participants reported being a current smoker (yes/no) and frequency of alcohol consumption (<twice a year/never, 1–2 times a month/every 2 months, 1–4 times per week, >5 times per week) were also included in models. Participants reported the frequency in which they engaged in vigorous, moderate and mild physical activity and we used these data to derive two possible categories reflecting regularity of physical activity: moderate/vigorous activity once a week or less, moderate/vigorous activity more than once a week. Doctor diagnosis of hypertension or use of anti-hypertensive medication was self-reported and these responses were combined with objective assessments taken at the nurse visit (hypertension defined as systolic blood pressure >140 and diastolic blood pressure >90) to generate a binary variable (yes/no); hypertension was included in models to predict the cardiometabolic diseases (i.e. CHD and diabetes/high blood glucose).

### Statistical analysis

Associations between variables were assessed using Pearson's correlations for continuous data and independent *t* tests for categorical data. Skewness and kurtosis values were within the acceptable range for all variables, and multicollinearity, assessed by examining variance inflation factors, was not apparent in any of the statistical models performed. Logistic regression was used to examine the relationship between baseline continuous adapted CES-D scores and sleep problem scores and incident morbidity reported between waves 5 and 7, controlling for covariates. Logistic regression was selected for two important reasons. First, creating a time variable using self-reported month and year of disease onset resulted in the loss of a large number of cases due to missing data. Second, creating a time variable based on the year of first reporting is arbitrary and is of little clinical value given participants were only assessed biennially. Depressive symptoms and sleep problems were entered into models simultaneously to assess the independent contribution of each variable over the other for predicting incident illness. Covariates were selected based on a priori knowledge and included age, sex, relationship status, wealth, smoking, BMI, frequency of alcohol consumption and regular physical activity. Hypertension was included in models to predict the cardiometabolic diseases: CHD and diabetes/high blood glucose. Variables were entered into models in two steps; first depressive symptoms and sleep were included and next all the covariates were entered into fully adjusted models. Separate models were performed to predict incident CHD, diabetes/high blood glucose, cancer and arthritis.

Secondary analyses were also performed looking at the interaction between depressive symptoms and sleep problems for predicting each incident illness. For these analyses, we created a binary variable (yes/no) using the standard cut-off on the CES-D of ⩾4. This represents a conservative estimate given our use of an adapted 7-item version of this scale and has been used by others (Steffick, 2000; Hamer *et al.*, 2009; Bell *et al.*, 2017). Sleep problems were transformed into a binary variable using a median split. Using these two binary variables, four categories were created, namely: low depressive symptoms/low sleep problems, high depressive symptoms/low sleep problems, low depressive symptoms/high sleep problems, and high depressive symptoms/high sleep problems. This categorical variable was entered into logistic regression models, using the low depressive symptoms/low sleep problem as the reference category, to predict incident CHD, diabetes/high blood glucose, cancer and arthritis, controlling for covariates.

Finally, sensitivity analyses were performed, to assess the effect of depressive symptoms and sleep symptoms on the incident disease when events reported within 1 wave of baseline were excluded from analyses. Results for all these models are presented as odds ratios (OR) with 95% confidence intervals (CI). All analyses were conducted using SPSS version 21. Two-tailed tests were used throughout and the significance level was set at *p* < 0.05, though exact significance levels are reported.

## Results

Table 1 displays the characteristics of the sample at baseline and follow-up, with regards to sociodemographic, behavioural, mood, sleep and incident chronic illness variables. The average age of the sample was 65.81 (±9.39) years, with a majority of participants falling within the 60–69 years age bracket. Approximately half of the participants were female and just over 70% were married or cohabiting with a partner. There was a good distribution of participants across each wealth quintile, from lowest to highest. In terms of the health and health behaviours of participants, the majority were non-smokers but did not engage in regular moderate or vigorous physical activity more than once a week. Just over half of the sample was hypertensive or on antihypertensive medication. While in general depressive symptom scores were low, 745 (10.1%) of the sample scored positively for elevated depressive symptoms using the adapted CES-D cut-off ⩾4. Chronic illnesses were prevalent in the sample (see Table 1), and in terms of incident illnesses (i.e. excluding those with the illness at baseline), the most common diagnoses at follow-up were arthritis followed by cancer and diabetes/high blood glucose. Depressive symptoms and sleep problem scores were significantly correlated (*r* = 0.35, *p* < 0.001).

**Table 1.** Demographic, clinical and biological characteristics of the sample (*N* = 7395)

Characteristic	Mean ± s.d. or *N* (%)
Baseline sociodemographics	
Age (years)	65.81 ± 9.39
Female	4049 (54.8)
Married/cohabiting	5209 (70.4)
Net financial wealth – quintiles	
1	1151 (15.6)
2	1427 (19.3)
3	1489 (20.1)
4	1590 (21.5)
5	1738 (23.5)
Baseline health and health behaviour	
Current smoker	977 (13.2)
Moderate/vigorous physical activity ⩾1 a week	2167 (29.3)
Alcohol consumption	
>5 times per week	1533 (20.7)
1–4 days per week	2663 (36.0)
1–2 times a month/every 2 months	1281 (17.3)
<Twice a year/never	1918 (25.9)
Body mass index (kg/m^2^)	28.25 ± 5.29
Hypertension	3954 (53.5)
Baseline mood and sleep problems	
CES-D adapted	0.99 ± 1.64
CES-D adapted ⩾4	745 (10.1%)
Sleep problems	2.28 ± 0.89
Follow-up incident morbidity	
CHD^a^	308 (6.1)
Diabetes/high blood glucose^b^	304 (8.4)
Cancer^c^	448 (8.6)
Arthritis^d^	695 (19.3)

a *N* = 5034.

b *N* = 3637.

c *N* = 5236.

d *N* = 3611.

### Depressive symptoms and sleep predicting incident CHD

In models to predict incident CHD, the sample size was 5034 with 308 incident cases over follow-up. In unadjusted models adapted CES-D score (OR 1.16, 95% CI 1.09–1.24, *p* < 0.001) but not sleep problems (OR 1.11, 95% CI 0.96–1.28, *p* = 0.147) was a significant predictor of incident CHD. In final models baseline adapted CES-D score (OR 1.11, 95% CI 1.04–1.20, *p* = 0.004) remained significantly related to an increased odds in CHD after controlling for covariates (see Table 2). Sleep problems were marginally non-significant (OR 1.15, 95% CI 1.00–1.34, *p* = 0.057). These results suggest for every 1 point increase in depressive symptoms there was an 11% increase in the odds of experiencing incident CHD.

**Table 2.** Baseline depressive symptoms predicting incident CHD at follow-up (*N* = 5034)

	OR	95% CI	*P*
Baseline depressive symptoms	1.11	1.04–1.20	0.004
Baseline sleep problems	1.15	1.00–1.34	0.057
Age (years)	1.07	1.05–1.08	<0.001
Sex^a^	0.50	0.39–0.65	<0.001
Relationship status^b^	0.68	0.51–0.91	0.009
Wealth			
1	Reference		
2	0.71	0.49–1.03	0.069
3	0.544	0.37–0.81	0.002
4	0.584	0.39–0.86	0.007
5	0.504	0.33–0.76	0.001
BMI (kg/m^2^)	1.02	1.00–1.04	0.090
Smoker^c^	0.66	0.46–0.94	0.023
Alcohol consumption			
>5 times per week	Reference		
1–4 days per week	1.00	0.71–1.40	0.977
1–2 times a month/every 2 months	1.01	0.67–1.53	0.952
<Twice a year/never	1.44	1.01–2.07	0.046
Physical activity^d^	0.71	0.52–0.96	0.026
Hypertension^e^	1.63	1.25–2.12	<0.001

a Female.

b Married/cohabiting.

c Smoker.

d Moderate/vigorous physical activity <1 a week.

e Not hypertensive.

N.B. Data represent the fully adjusted model.

Secondary fully adjusted analyses to examine the interaction between depressive symptoms and sleep showed that compared with those with low depressive symptoms/low sleep problems (*n* = 2531) there was an increased odds in future CHD incidence associated with each depressive symptoms/sleep problems combination. Specifically, compared with the reference group, the high depressive symptoms/low sleep problems group (*n* = 82) (OR 3.73, 95% CI 2.00–6.89, *p* < 0.001), the low depressive symptoms/high sleep problems (*n* = 2069) (OR 1.36, 95% CI 1.04–1.76, *p* = 0.023) and the high depressive symptoms/high sleep problems group (*n* = 352) (OR 1.74, 95% CI 1.13–2.68, *p* = 0.011) were associated with an increase in the odds of CHD at 6 year follow-up.

Sensitivity analyses were also performed, removing participants who developed CHD within 1 wave of baseline. The sample size for these analyses reduced to 4858 with 132 incident cases of CHD. The results of the main analysis were attenuated with depressive symptoms (OR 1.11, 95% CI 1.00–1.24, *p* = 0.055) and sleep problems (OR 1.05, 95% CI 0.85–1.30, *p* = 0.666) associated with a non-significant increase in odds of incident CHD. The interaction analyses were also attenuated in these sensitivity analyses (results not reported).

### Depressive symptoms and sleep predicting incident diabetes/high blood glucose

In models to predict incident diabetes/high blood glucose, the sample size was 3637 with 304 incident cases over follow-up. In unadjusted models, adapted CES-D score (OR 1.22, 95% CI 1.14–1.30, *p* < 0.001) but not sleep problems (OR 1.02, 95% CI 0.88–1.18, *p* = 0.787) predicted incident diabetes/high blood glucose. In fully adjusted models depressive symptoms (OR 1.13, 95% CI 1.04–1.22, *p* = 0.002) remained a significant predictor of incident diabetes/high blood glucose. Sleep problems were not a significant predictor in fully adjusted models (OR 0.99, 95% CI 0.85–1.16, *p* = 0.922). See Table 3 for these results. These results suggest that for every 1 point increase in depressive symptoms there was a 13% increase in the odds of experiencing incident diabetes/high blood glucose. Secondary analyses revealed no significant interaction effects between depressive symptoms and sleep problems (results not reported).

**Table 3.** Baseline depressive symptoms predicting incident diabetes/high blood glucose at follow-up (*N* = 3637)

	OR	95% CI	*P*
Baseline depressive symptoms	1.13	1.04–1.22	0.002
Baseline sleep problems	0.99	0.85–1.16	0.922
Age (years)	1.03	1.02–1.05	<0.001
Sex^a^	0.55	0.42–0.72	<0.001
Relationship status^b^	0.90	0.66–1.21	0.474
Wealth			
1	Reference		
2	0.79	0.54–1.17	0.240
3	0.67	0.45–1.01	0.054
4	0.53	0.34–0.81	0.004
5	0.60	0.39–0.92	0.020
BMI (kg/m^2^)	1.15	1.12–1.17	<0.001
Smoker^c^	0.54	0.38–0.77	0.001
Alcohol consumption			
>5 times per week	Reference		
1–4 days per week	1.08	0.75–1.56	0.672
1–2 times a month/every 2 months	1.61	1.07–2.42	0.023
<Twice a year/never	1.23	0.82–1.83	0.317
Physical activity^d^	0.57	0.42–0.79	0.001
Hypertension^e^	1.88	1.43–2.46	<0.001

a Female.

b Married/cohabiting.

c Smoker.

d Moderate/vigorous physical activity <1 a week.

e Not hypertensive.

N.B. Data represent the fully adjusted model.

Sensitivity analyses were also performed, removing participants who developed diabetes/high blood glucose within 1 wave of baseline. The sample size for these analyses reduced to 3551 with 218 incident cases of diabetes/high blood glucose. The results of the main analysis were replicated with depressive symptoms (OR 1.10, 95% CI 1.01–1.21, *p* = 0.037) but not sleep problems (OR 0.96, 95% CI 0.80–1.16, *p* = 0.691) associated with a significant increase in odds of incident diabetes/high blood glucose.

### Depressive symptoms and sleep predicting incident cancer

In models to predict incident cancer, the sample size was 5236 with 448 incident cases over follow-up. In unadjusted models, neither adapted CES-D score (OR 1.06, 95% CI 0.99–1.12, *p* = 0.088) nor sleep problems (OR 1.09, 95% CI 0.97–1.22, *p* = 0.159) predicted incident cancer 6 years later. Similarly, in final models neither depressive symptoms (OR 1.05, 95% CI 0.98–1.12, *p* = 0.137) nor sleep problems (OR 1.11, 95% CI 0.98–1.25, *p* = 0.096) were a significant predictor of incident cancer, independent of covariates.

However, secondary fully adjusted analyses to examine the interaction between depressive symptoms and sleep showed that compared with those with low depressive symptoms/low sleep problems (*n* = 2584) those with high depressive symptoms/high sleep problems group (*n* = 373) were at increased odds of incident cancer (OR 1.67, 95% CI 1.17–2.39, *p* = 0.004) suggesting a combined deleterious effect for future cancer risk with a 67% increase in odds for this group compared with those low in depressive symptoms and sleep problems. Compared with the reference group, neither the high depressive symptoms/low sleep problems (*n* = 87) (OR 0.79, 95% CI 0.34–1.85, *p* = 0.584) nor the low depressive symptoms/high sleep problems (*n* = 2192) (OR 1.06, 95% CI 0.86–1.31, *p* = 0.597) group were at increased odds of incident cancer at follow-up (see Table 4).

**Table 4.** Elevated depressive symptoms and elevated sleep problems predicting incident cancer at follow-up (*N* = 5236)

	OR	95% CI	*p*
adapted CES-D × sleep interaction			
Low depressive symptoms/low sleep problems	Reference		
High depressive symptoms/low sleep problems	0.79	0.34–1.85	0.584
Low depressive symptoms/high sleep problems	1.06	0.86–1.31	0.597
High depressive symptoms/high sleep problems	1.67	1.17–2.39	0.004
Age (years)	1.04	1.03–1.05	<0.001
Sex^a^	0.89	0.72–1.09	0.246
Relationship status^b^	0.94	0.74–1.19	0.585
Wealth			
1	Reference		
2	1.02	0.71–1.45	0.930
3	1.19	0.84–1.70	0.321
4	1.21	0.85–1.72	0.290
5	1.08	0.75–1.55	0.698
BMI (kg/m^2^)	1.01	0.99–1.03	0.289
Smoker^c^	0.82	0.60–1.11	0.201
Alcohol consumption			
>5 times per week	Reference		
1–4 days per week	0.97	0.75–1.26	0.819
1–2 times a month/every 2 months	0.83	0.59–1.15	0.250
<Twice a year/never	0.87	0.64–1.178	0.362
Physical activity^d^	0.79	0.62–0.99	0.040

a Female.

b Married/cohabiting.

c Smoker.

d Moderate/vigorous physical activity <1 a week.

N.B. Data represent the fully adjusted model.

Sensitivity analyses were also performed, removing participants who developed cancer within 1 wave of baseline. The sample size for these analyses reduced to 5054 with 266 incident cases of cancer. Analyses looking at the interaction between depressive symptoms and sleep problems were confirmed in these sensitivity analyses, with only those with high depressive symptoms/high sleep problems group (*n* = 358) at increased odds of incident cancer (OR 1.86, 95% CI 1.21–2.85, *p* = 0.005) compared with the low depressive symptoms/low sleep problems reference group (*n* = 2501).

### Depressive symptoms and sleep predicting incident arthritis

In models to predict incident arthritis, the sample size was 3611 with 695 incident cases over follow-up. In unadjusted models, adapted CES-D score (OR 1.12, 95% CI 1.06–1.18, *p* < 0.001) and sleep problems (OR 1.19, 95% CI 1.08–1.32, *p* = 0.001) were both independent predictors of incident arthritis. In final models, baseline adapted CES-D score (OR 1.10, 95% CI 1.04–1.16, *p* = 0.002) and sleep problems (OR 1.14, 95% CI 1.02–1.26, *p* = 0.019) remained significant (see Table 5). These results suggest that for every 1-point increase in depressive symptoms there was a 10% increase in the odds and with every 1-point increase in sleep problems there was a 14% increase in the odds of incident arthritis.

**Table 5.** Baseline depressive symptoms and sleep problems predicting incident arthritis at follow-up (*N* = 3611)

	OR	95% CI	*p*
Baseline depressive symptoms	1.10	1.04–1.16	0.002
Baseline sleep problems	1.14	1.02–1.26	0.019
Age (years)	1.02	1.01–1.03	<0.001
Sex^a^	1.50	1.26–1.79	<0.001
Relationship status^b^	1.12	0.91–1.37	0.285
Wealth			
1	Reference		
2	0.75	0.55–1.02	0.063
3	0.67	0.50–0.91	0.011
4	0.66	0.48–0.89	0.007
5	0.62	0.45–0.84	0.002
BMI (kg/m^2^)	1.05	1.04–1.07	<0.001
Smoker^c^	1.25	0.94–1.66	0.125
Alcohol consumption			
>5 times per week	Reference		
1–4 days per week	1.07	0.85–1.35	0.584
1–2 times a month/every 2 months	0.91	0.68–1.21	0.495
<Twice a year/never	1.00	0.76–1.31	0.989
Physical activity^d^	1.07	0.89–1.29	0.477

a Female.

b Married/cohabiting.

c Smoker.

d Moderate/vigorous physical activity <1 a week.

N.B. Data represent the fully adjusted model.

Secondary fully adjusted analyses to examine the interaction between depressive symptoms and sleep showed that compared with those with low depressive symptoms/low sleep problems (*n* = 1983) those with low depressive symptoms/high sleep problems (*n* = 1387) (OR 1.32, 95% CI 1.11–1.58, *p* = 0.002) were at increased odds of incident arthritis. There was also a significant detrimental effect for the high depressive symptoms/high sleep problems group (*n* = 186) (OR 1.86, 95% CI 1.30–2.64, *p* = 0.001) compared with those low in these symptoms. However, compared with the reference group, the high depressive symptoms/low sleep problems group (*n* = 55) (OR 1.77, 95% CI 0.97–3.27, *p* = 0.065) were not at increased odds of incident arthritis.

Sensitivity analyses were also performed, removing participants who developed arthritis within 1 wave of baseline. The sample size for these analyses reduced to 3326 with 410 incident cases of arthritis. Results from the main analysis corroborated the significant relationship between sleep problems (OR 1.18, 95% CI 1.03–1.35, *p* = 0.014) and future arthritis. However, depressive symptoms (OR 1.07, 95% CI 1.00–1.16, *p* = 0.056) were marginally non-significant. Sensitivity analyses looking at the interaction between depressive symptoms and sleep problems showed the same pattern of findings as was found in the main analyses, with those with low depressive symptoms/high sleep problems (*n* = 1266) (OR 1.37, 95% CI 1.10–1.71, *p* = 0.006) and those with high depressive symptoms/high sleep problems (*n* = 163) (OR 1.94, 95% CI 1.26–3.00, *p* = 0.003) being associated with an increased odds of future arthritis compared with the low depressive symptoms/low sleep problems reference group (*n* = 1850).

## Discussion

This study sought to examine the combined effect of depressive symptoms and sleep problems for incident physical illness onset up to 6 years later in a nationally representative cohort of adults aged 50 years and older. The principle findings showed that depressive symptoms predicted incident CHD and diabetes/high blood glucose independent of sleep problems. Both depressive symptoms and sleep problems predicted incident arthritis even after taking into consideration a wide range of sociodemographic, behavioural and clinical factors. No main effects were evident for predicting cancer, however, a combination of elevated sleep problems and elevated depressive symptoms was associated with increased odds. Results for diabetes/high blood glucose and cancer, but not CHD, were replicated in sensitivity analyses excluding participants who reported disease within 1 wave of baseline. Findings for arthritis were partly replicated in sensitivity analyses though depressive symptoms were no longer significant in the main effects model. These null findings for CHD and arthritis in our sensitivity analyses could possibly indicate confounding by cases diagnosed shortly after baseline or else they could have been caused by a reduction in the sample size affecting the ability to detect an effect (e.g. cases reduced from 308 to 132 in the CHD analyses).

Our main finding was that depressive symptoms and sleep problems were significantly associated with disease onset in ELSA, but that the pattern of results varied according to the physical illness in question. The link between depression and sleep problems is now well-established (Benca and Peterson, 2008) though the pathways linking them are not unidirectional (Staner, 2010). While disturbed sleep is one of the key diagnostic features of MDD, sleep disturbance is also known to precede the onset of depression (Roberts *et al.*, 2000; Almeida *et al.*, 2011; Baglioni *et al.*, 2011). We, therefore, entered depressive symptoms and sleep scores simultaneously into our models in order to look at the independent effect of each factor while holding the other constant. In fully adjusted models depressive symptoms but not sleep problems predicted the cardiometabolic disorders, namely CHD and diabetes/high blood glucose, however, both depressive symptoms and sleep problems were significant predictors after taking into account multiple confounders in models to predict arthritis. A combination of elevated sleep problems and elevated depressive symptoms was a significant predictor of incident cancer in fully adjusted models. The interaction between depressive symptoms and sleep problems was also apparent for CHD and arthritis. These results add credence to research supporting the independent effect of depressive symptoms and sleep problems for health outcomes.

Many studies have looked at the role of depression for disease risk, for example, depression has been shown to predict the onset of CHD (Kuper *et al.*, 2002; Rugulies, 2002; Nicholson *et al.*, 2006), diabetes (Mezuk *et al.*, 2008) and cancer (Chida *et al.*, 2008). Indeed, previously published work using ELSA has supported the link between depression and risk of diabetes (Demakakos *et al.*, 2010). The magnitude of the effect of depressive symptoms was similar in our models to predict both CHD and diabetes/high blood glucose, with an 11% and 13% increase in the odds of disease onset respectively, for every 1-point increase in the adapted CES-D score. In arthritis models, a 1-point increase in sleep problems was associated with a larger increase in odds compared with a 1-point increase in depressive symptoms (14% *v.* 10%). Direct comparisons with previous studies are circumvented by differences in measurement and analysis. For example, Nicholson and colleagues (Nicholson *et al.*, 2006) found depression was associated with a relative risk of CHD of 1.90 (95% CI = 1.49–2.42) in adjusted studies; Mezuk and colleagues (Mezuk *et al.*, 2008) found that depression was associated with a relative risk of diabetes of 1.60 (95% CI = 1.37–1.88). The evidence of depressive symptoms for arthritis onset is lacking, though it is thought to confer increased risk (Patten *et al.*, 2008). For example, one study found that the incidence of rheumatoid arthritis was higher in depressed than non-depressed individuals (adjusted hazard ratio = 1.65, 95% CI 1.41–1.77) (Lu *et al.*, 2016).

However, these studies have not taken into account the role of sleep problems. Sleep problems have been cross-sectionally associated with many physical illnesses (Foley *et al.*, 2004; Koyanagi *et al.*, 2014), but few studies have looked at their relationship with disease onset using prospective analyses. There is some evidence sleep problems play a role in CHD mortality in males (Mallon *et al.*, 2002) and as a risk factor for incident MI (Schwartz *et al.*, 1998). There is also some evidence to suggest that sleep problems (specifically difficulties initiating and maintaining sleep) are predictive of incident diabetes in men (Mallon *et al.*, 2005). Sleep problems have not been investigated with regards to cancer onset, though there is some evidence for the role of sleep duration (Gu *et al.*, 2016).

The combined effect of sleep problems and depressive symptoms has been little studied in relation to disease onset, and work has tended to focus on cardiometabolic diseases (Mezick *et al.*, 2011). To the best of our knowledge, our study is the first to systematically investigate the longitudinal effect of sleep problems and depressive symptoms for disease onset, across a variety of disease types. Our interaction analyses partly support the conclusions of Mezick *et al*. (2011) who suggest sleep problems to be a risk factor for CVD, independent of depression, but that inconclusive evidence exists for metabolic diseases (i.e. diabetes). We have added to this body of work showing that sleep problems may also play a role in the onset of arthritis. Further work is needed to corroborate the null findings for diabetes/high blood glucose that we report here for sleep, perhaps using a longer length of follow-up and further distinguishing between insulin resistance and diabetes.

We also examined interaction effects between depressive symptoms and sleep problems, to investigate whether there was any increased risk involved in having one complaint rather than the other, or whether having both was particularly detrimental to health. We found that the interaction between depressive symptoms and sleep was important in predicting incident CHD, cancer and arthritis. Specifically, high depressive symptoms/low sleep problems, low depressive symptoms/high sleep problems and high depressive symptoms/high sleep problems were all predictive of incident CHD, suggesting a role for both symptom profiles in cardiac disease. In models predicting incident cancer, only the combination of high depressive symptoms/high sleep problems was a significant predictor, indicating that having elevations in both symptom profiles was particularly health damaging. Finally, low depressive symptoms/high sleep problems and high depressive symptoms/high sleep problems were found to significantly predict arthritis onset. Overall, these results highlight the importance of considering the overlap that depression has with somatic complaints, such as sleep disturbance, as well as considering them as separate constructs.

One possible way to reconcile the two constructs is to turn to the literature on vital exhaustion. Vital exhaustion is commonly defined as symptoms of excessive tiredness, increased irritability and a sense of demoralisation (Appels *et al.*, 1987). Vital exhaustion has been shown to be predictive of heart disease onset (Prescott *et al.*, 2003), but there is less evidence for its role in cancer (Bergelt *et al.*, 2005). Although depression and vital exhaustion are generally understood to be separate constructs (Kopp *et al.*, 1998), in a recent study this was only observed in men and not women (Lindeberg *et al.*, 2012). Another study used principal component analyses on questionnaire responses of 528 MI patients on the Beck Depression Inventory (BDI) and Maastricht Questionnaire (a measure of vital exhaustion) (Vroege *et al.*, 2012). These authors showed strong conceptual overlap between the two scales, with all but two items from the Maastricht Questionnaire loading on the somatic/affective dimension of the BDI. Moreover, this somatic/affective dimension was able to predict recurrent events, but the cognitive/affective dimension was not. Due to these disparate findings, more research is needed to delineate the independent effect of vital exhaustion over and above the constructs of depression and sleep disturbance, on physical disease risk.

There are several possible mechanisms for the link between sleep and disease onset. Interestingly these pathways are similar to those proposed to link depression to physical illness (Brown *et al.*, 2004; Dantzer *et al.*, 2008). Primary biological systems thought to be implicated include the role the neuroendocrine system and in particular, cortisol (Meerlo *et al.*, 2008). Changes to the neuroendocrine system have been observed after sleep loss, although the findings are inconsistent, with some studies reporting a slight elevation in cortisol in response to sleep deprivation (Spiegel *et al.*, 1999; Chapotot *et al.*, 2001), while others have reported no changes or even small decrements in cortisol levels (Akerstedt *et al.*, 1980; Follenius *et al.*, 1992). Depression has long been associated with increases in cortisol output (Zunszain *et al.*, 2011). Modulation of cortisol responses may be particularly relevant to inflammatory diseases such as CHD and arthritis since cortisol is known to be involved in regulation of the immune system (Maier and Watkins, 1998). Both disturbed and curtailed sleep have indeed been shown to increase low-grade inflammation directly (Irwin, 2015) and depression is known to be associated with pro-inflammatory cytokines such as interleukin-6 (Howren *et al.*, 2009). Recent evidence suggests that the relationship between depression and inflammation is symptom-specific, with the fatigue-type symptoms being most strongly associated with C-reactive protein, but this effect was absent in those using antidepressant medication (White *et al.*, 2017). While such studies allude to the anti-inflammatory effects of antidepressant medication, we were unable to take this into account in our analyses since this information was only collected on a small number of participants who reported a positive doctor diagnosis of depression in ELSA. Sleep is known to change with age (Ohayon *et al.*, 2004), therefore, it is also plausible that sleep effects processes involved in the biology of ageing such as telomere length (Jackowska *et al.*, 2012; Tempaku *et al.*, 2015). More work is needed to tease out the exact pathways by which sleep and depression operate to affect future disease onset. Our findings suggest sleep problems and depressive symptoms precede the onset of physical illness, though causality cannot be ascertained from our work since sub-clinical measures of disease were not recorded in ELSA. We attempted to control for this effect in sensitivity analyses, though longer-term studies are needed to corroborate our findings.

Our work had a number of strengths. First, our use of nationally representative data, providing us with a large cohort on which to test our research questions, improves the generalisability of our findings. We controlled for a wide range of confounders that might otherwise predict physical illness onset and were still able to demonstrate an effect. Moreover, the prospective nature of ELSA allowed us to conduct longitudinal analyses, looking at the effect of baseline depressive symptoms and sleep problems for future disease risk. Importantly, looking at multiple disease outcomes increases the salience of our findings, showing these factors are likely to be important across a variety of illnesses. However, a number of weaknesses must also be noted. First, was our reliance on self-report data of physical illness which may be prone to recall bias and reporting errors. We tried to circumvent this limitation by using objective data where possible (i.e. blood pressure readings and HbA1c values). Second, due to missing data regarding the date of illness onset, we were unable to use survival analysis techniques. Therefore, our study is limited to observing cases of incident disease and cannot make any claims about the rate of onset. Third, we investigated the role of depressive symptoms and not MDD, therefore our results are not readily generalisable to those with clinical episodes of depression. Fourth, compared with those excluded from analyses, those included represented a more affluent and healthier subset of individuals, which may have lead us to underestimate some of the effects. However, we believe this to be congruent with the fact we have restricted our sample to those free from physical illness at baseline. Finally, we were unable to take into account objective measures of sleep, use of sleep medication, or clinical diagnoses such as sleep apnoea, since these were not collected in ELSA. However, our findings do suggest an effect of self-reported sleep on future disease risk, highlighting the importance of the subjective experience of sleep for future wellbeing.

In conclusion, we found that depressive symptoms predicted incident CHD and diabetes/high blood glucose, depressive symptoms and sleep problems predicted incident arthritis and a combination of elevated depressive symptoms and elevated sleep problems predicted incident cancer relative to those low in these symptoms 6 years later in a nationally representative cohort of adults aged 50 years and older. While mechanisms of the effect are unclear, our findings point to the importance of simultaneously taking into account somatic and affective experiences when looking across a variety of different physical illnesses.
